# Clinical profile and risk factors for respiratory failure in children with *Mycoplasma pneumoniae* infection

**DOI:** 10.17305/bb.2024.11641

**Published:** 2025-01-22

**Authors:** Yanfei Wang, Limin Huang, Junjie Qian, Kelei Deng, Zihao Yang, Zhenjie Chen, Wei Li, Linhua Tan

**Affiliations:** 1 Department of Surgical ICU, Children’s Hospital, Zhejiang University School of Medicine, National Clinical Research Center for Child Health, Hangzhou, China; 2 Department of Nephrology, Children’s Hospital, Zhejiang University School of Medicine, National Clinical Research Center for Child Health, Hangzhou, China; 3 Department of Pediatric ICU, Children’s Hospital, Zhejiang University School of Medicine, National Clinical Research Center for Child Health, Hangzhou, China; 4 Department of Laboratory, Children’s Hospital, Zhejiang University School of Medicine, National Clinical Research Center for Child Health, Hangzhou, China

**Keywords:** *Mycoplasma pneumoniae*, MP, respiratory failure, clinical disease prediction model

## Abstract

*Mycoplasma pneumoniae* (MP) is a common cause of community-acquired pneumonia (CAP) in children and can lead to severe complications, including respiratory failure. A retrospective analysis of 2084 children diagnosed with CAP and treated in our hospital from January 2022 to January 2023 was conducted. A comprehensive dataset of patient demographics, clinical symptoms, and laboratory findings was initially assembled. Subsequent statistical analyses were carried out to elucidate the clinical characteristics of MP pneumonia (MPP) in children. Additionally, the study identified high-risk factors for respiratory failure in the context of MPP. Among the hospitalized MPP cases, 15.8% progressed to respiratory failure. Statistical analysis identified D-dimer level as a significant risk factor for respiratory failure in children with MPP. A predictive model was developed using D-dimer levels, yielding an area under the curve (AUC) of 0.818 with a cutoff value of 1.015 mg/L. The model demonstrated a sensitivity of 62.4% and a specificity of 91.3%, proving effective in predicting respiratory failure caused by MPP. Respiratory failure remains a critical complication in children with MPP, and D-dimer levels serve as a key predictive risk factor. Vigilant monitoring of coagulation function, particularly D-dimer levels, is essential for the early identification of patients at risk of developing respiratory failure in MPP cases.

## Introduction

Community-acquired pneumonia (CAP) is a common disease that can become life-threatening in severe cases. Despite significant advancements in treatment, CAP remains a leading cause of morbidity and mortality worldwide. Mycoplasma pneumoniae (MP) is one of the most frequent causes of CAP [1], accounting for 40% of cases in children. Compared with other pathogens, patients with CAP caused by atypical bacteria (mainly MP) often experience a longer disease course but lower severity overall [2]. The annual incidence of sporadic MP pneumonia (MPP) in children and adolescents is 3–4 per 1000 individuals, with rates increasing significantly during outbreaks, which occur every 4–7 years [3]. Additionally, 18% of pediatric MPP cases require hospitalization [4]. MP infection typically presents with mild, self-limiting symptoms. Common clinical manifestations include fever, cough, sore throat, shortness of breath, and, in some cases, dyspnea. However, patients of all ages can develop severe pulmonary or extrapulmonary complications. Pulmonary complications may include lung abscess, obliterative bronchiolitis, or respiratory failure [5], while extrapulmonary complications can involve various organ systems. For example, MP infection can lead to myocarditis in the heart, causing arrhythmias or heart failure [6]. In the liver, it can result in hepatitis, manifesting as elevated liver enzyme levels or jaundice [7]. It can also significantly impact the nervous system (e.g., causing meningitis or encephalitis), the hematological system (e.g., hemolytic anemia), the skin and mucous membranes (e.g., rash and mucositis) [8], and the musculoskeletal system (e.g., arthritis) [9]. These diverse complications are primarily due to complex immunopathological mechanisms triggered by MP. The pathogen activates the immune response, which leads to the release of inflammatory mediators and autoantibodies. These immune reactions interact with self-antigens in various organs and tissues, ultimately causing tissue damage and functional impairment [10]. Consequently, MPP not only consumes significant medical resources but also imposes a substantial healthcare burden [11]. The increasing misuse of antibiotics has contributed to a high degree of genetic variability in MP, leading to rising macrolide resistance both domestically and internationally. This growing resistance makes treatment more difficult. Additionally, as the incidence of severe and refractory MPP increases, notable changes in its pathogenic mechanisms have been observed. MP triggers an immune response and induces cytokine release, initiating an inflammatory process characterized by the accumulation of fibrinous exudate in the alveolar cavity. This increases the likelihood of severe lung lesions, with lobar pneumonia being a prominent manifestation [12, 13]. Lobar pneumonia, a common pathological form of MPP, can lead to serious complications, including atelectasis, pneumothorax, pleural effusion, and even respiratory failure [14].

Respiratory failure is a common concern in pediatric emergency departments and intensive care units. It can result from various conditions, including acute parenchymal or obstructive lung diseases, congestive heart failure, neuromuscular disorders, or diseases affecting the control of the ventilatory center [15]. Severe infection with MP is another potential trigger for respiratory failure, often leading to significant complications. Initially, children may present with symptoms, such as rapid and labored breathing. As the condition progresses, hypoxia and carbon dioxide buildup can cause tissue and organ dysfunction, potentially resulting in complications such as acidosis [1]. In severe cases, respiratory failure can increase cardiac workload and heighten the risk of cardiovascular events [9]. This condition is life-threatening and demands prompt intervention and treatment [16]. Understanding the clinical characteristics and risk factors associated with respiratory failure in children with MPP is of significant clinical importance. This study seeks to identify high-risk factors for predicting respiratory failure in these children by analyzing clinical data from MPP inpatients at our hospital in recent years. The goal is to aid clinicians in early detection, timely treatment, and improved patient outcomes.

## Materials and methods

### Study population

This study included patients diagnosed with MPP, admitted to the Children’s Hospital of Zhejiang University School of Medicine from January 2022 to January 2023, aged 28 days to 18 years. Diagnosis was based on: (1) acute respiratory symptoms, such as fever, cough, shortness of breath and/or difficulty breathing; (2) chest X-ray or CT shows signs of inflammatory infiltration, which mainly consists of patchy shadows, lobar or segmental consolidation, and interstitial changes. In some cases, pleural effusion may be present. There may or may not be abnormal lung sounds such as wheezing or moist rales; (3) serological detection of positive MP-IgM (A single serum sample shows an MP-IgM antibody titer of ≥ 1:160, or the antibody titer in the convalescent-phase serum has increased by four times or more) and MP RNA (The nucleic acid of MP in clinical specimens was detected by quantitative polymerase chain reaction (PCR), and the test result was positive) in nasopharyngeal swabs. Exclusion criteria included: (1) underlying diseases (malignant tumors, congenital respiratory malformations, immunodeficiency); (2) multiple pathogen infections or infection foci; (3) recovery phase of MPP (disease duration >4 weeks, stable temperature >1 week, imaging improvement) [11, 17].

Subsequently, all enrolled children were divided into two groups based on whether they experienced respiratory failure during hospitalization: the MPP with respiratory failure group and the non-respiratory failure group. Respiratory failure was diagnosed based on blood gas abnormalities, specifically a partial pressure of oxygen (PaO_2_) <60 mmHg, a partial pressure of carbon dioxide (PaCO_2_) >55 mmHg, and an oxygen saturation level (SaO_2_) <90% [16].

Retrospective clinical data were meticulously collected and evaluated from patient records. These data included fundamental details, such as age, gender, clinical symptoms, signs, pulmonary and extrapulmonary complications, fever duration, and length of hospital stay (LOS), along with a wide range of laboratory parameters. The laboratory parameters, continuously monitored during hospitalization, encompassed white blood cell count (WBC), neutrophil percentage, C-reactive protein (CRP), procalcitonin (PCT), lactate dehydrogenase (LDH), D-dimer, and other relevant clinical indicators. The final dataset reflected the period when each patient’s condition was at its most severe. This determination was based on a comprehensive evaluation of overall trends and the most critical values of these indicators, aiming to capture the disease’s most pronounced manifestations and its impact on the patient’s health. Laboratory tests were conducted in the hospital’s clinical laboratory. Cytokines, immunoglobulins, and D-dimer levels were measured using enzyme-linked immunosorbent assay (ELISA) kits. MP antibodies were detected via the colloidal gold method for MP antigen (manufactured by Hangzhou Genesis Biodetection & Biocontrol Co., Ltd., China). Nucleic acid detection of 13 respiratory pathogens, including MP, was performed using the fluorescence PCR-capillary electrophoresis method with the Respiratory Pathogen Multiplex Detection System (Health BioMed Co., China). PCT levels were quantified via immunofluorescence assay. Complement components were measured using immunochemical methods. Cell phenotypic analysis was conducted through flow cytometry. The erythrocyte sedimentation rate (ESR) was measured with an ESR analyzer, while complete blood counts were obtained via an automated cell counter. Coagulation profiles were assessed using an automated coagulation analyzer, and biochemical parameters were measured with an automated biochemical analyzer.

### Ethical statement

This study was conducted in accordance with the Helsinki Declaration and approved by the Ethics Committee of Children’s Hospital of Zhejiang University School of Medicine (2022-IRB-193). Given the retrospective nature of the study, informed consent was waived.

### Statistical analysis

Data were processed using SPSS 26.0 and R version 4.4.1 (specifically, the “glmnet” and “pROC” packages). The K-S test was employed to assess the normality of the data. Quantitative data that followed a normal distribution are presented as the mean ± standard deviation (x ± s) and were compared using independent sample *t*-tests. Non-normally distributed data are shown as the median and interquartile range (median, interquartile range) and were compared using the Mann–Whitney *U* test. Count data are expressed as percentages (%) and analyzed using Fisher’s exact test or chi-square test. Spearman’s rank-order correlation was employed for correlation analysis. High-risk factors for respiratory failure in MPP patients were identified through logistic regression and the Least Absolute Shrinkage and Selection Operator (Lasso) method. A clinical prediction model was constructed, and its predictive probabilities were validated using ROC curve analysis and Bootstrap resampling. An area under the curve (AUC) >0.5 indicated predictive value. Statistical significance was set at *P* < 0.05, with a 95% confidence interval.

## Results

### Baseline information

**Figure 1.**
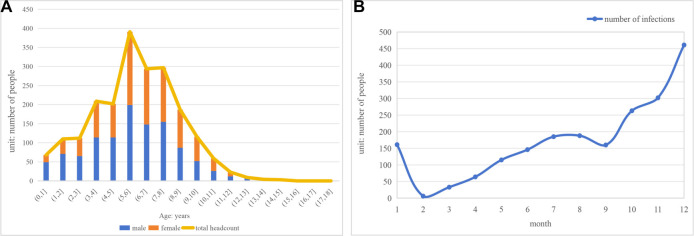
**The overall clinical condition of children with MPP.** (A) Bar chart of age and sex distribution among hospitalized children with MPP; (B) Trend chart of monthly positive cases of hospitalized children with MPP at the hospital from 2022 to 2023. MPP: Mycoplasma pneumoniae pneumonia.

In this study, 2084 children with MPP were selected from the hospital’s pneumonia inpatients. The median age was six years, and 52.83% were male. MP was detected throughout 2023, with the highest MPP incidence occurring in December (Figure 1). Based on respiratory failure criteria, the children were divided into two groups: 329 (15.8%) with MPP and respiratory failure, and 1755 (84.2%) with MPP only. An analysis of clinical characteristics (Table 1) revealed that the most common symptoms among children with MPP were cough (99.5%) and fever (94.8%), followed by sputum production (88.5%). Less frequent symptoms included vomiting (7.4%) and diarrhea (2.0%). There were no significant differences between the two groups in the incidence of fever, cough, sputum production, vomiting, or diarrhea (*P* < 0.05). However, children with respiratory failure were younger, had higher peak fevers, and experienced longer durations of fever and hospital stays (*P* < 0.05). Gender distribution was not significantly different between the groups (*P* > 0.05). Treatment analysis showed that children with respiratory failure were more likely to receive steroids and combination therapy with third-line antibiotics (e.g., carbapenems) (*P* < 0.05). In contrast, macrolide antibiotics alone were more commonly used in children without respiratory failure. All children with MPP and respiratory failure received oxygen therapy: 79.94% via nasal cannula (average duration 3.9 ± 2.9 days), 20.36% via mask (2.9 ± 2.1 days), and 1.82% via mechanical ventilation (11.0 ± 11.2 days). Additionally, 5.78% required ICU admission, with an average stay of 11.3 ± 7.1 days. About 42.2% of children with respiratory failure required fiberoptic bronchoscopy, significantly higher than the 22.9% of those without respiratory failure (*P* < 0.05).

**Table 1:** Comparison of clinical characteristics in pediatric MPP with and without respiratory failure

**Characteristic**	**MPP with respiratory failure**	**MPP without respiratory failure**	**Chi-square value**	***P* value**
*Clinical features*				
Cough (*n*, %)	327 (99.4%)	1747 (99.5%)	0.134	0.714
Expectoration (*n*, %)	291 (88.4%)	1554 (88.5%)	0.003	0.960
Vomit (*n*, %)	26 (7.9%)	129 (7.4%)	0.123	0.726
Diarrhea (*n*, %)	9 (2.7%)	32 (1.8%)	1.195	0.274
Fever (*n*, %)	311 (94.5%)	1665 (94.9%)	0.066	0.797
T max, ^∘^C^a^	39.6 (39.0,40.0)	39.5 (39.0,40.0)	−2.158	0.031
Fever course, day^a^	9 (6,10)	8 (6,9)	−4.032	<0.001
Length of hospital stay, day^a^	9 (6,12)	6 (4,8)	−14.444	<0.001
Age, year^a^	5.9 (3.4,8.0)	6.0 (4.4,8.0)	−4.014	<0.001
Sex, male (*n*, %)	929 (52.9%)	172 (52.3%)	0.048	0.827
Steroids usage (*n*, %)	269 (81.76%)	842 (47.98%)	127.060	<0.001
*Antibiotic usage*				
Macrolides (*n*, %)	71 (21.58%)	555 (31.62%)	13.298	<0.001
Macrolides + Penicillin (*n*, %)	44 (13.37%)	243 (13.85%)	0.052	0.820
Macrolides + Cephalosporin (*n*, %)	134 (40.73%)	683 (38.92%)	0.382	0.537
Others^b^ (*n*, %)	80 (24.32%)	274 (15.61%)	14.884	<0.001
*Respiratory support therapy*				
Nasal cannula oxygen therapy (days, %)	3.9 ± 2.9 (79.94%)	/	/	/
Mask oxygen therapy (days, %)	2.9 ± 2.1 (20.36%)	/	/	/
Mechanical ventilation therapy (days, %)	11.0 ± 11.2 (1.82%)	/	/	/
ICU length of hospital stay (days, %)	11.3 ± 7.1 (5.78%)	/	/	/
Fiber bronchoscopy (*n*, %)	139 (42.2%)	424 (22.9%)	54.923	<0.001
*Complications*				
Pulmonary consolidation (*n*, %)	149 (45.30%)	460 (26.20%)	48.756	<0.001
Pleural effusion (*n*, %)	141 (42.90%)	305 (17.40%)	106.920	<0.001
Liver function impairment (*n*, %)	42 (12.80%)	62 (3.50%)	49.817	<0.001
Myocardial injury (*n*, %)	7 (2.10%)	22 (0.70%)	6.394	0.011
Gastrointestinal complications (*n*, %)	56 (17.02%)	329 (18.75%)	0.547	0.459
Nonspecific rash (*n*, %)	39 (11.85%)	191 (10.88%)	0.266	0.606
Neurological complications (*n*, %)	10 (3.04%)	12 (0.68%)	15.007	<0.001

^a^This portion of data is continuous. In the analysis process, the Mann–Whitney *U* test is employ; ^b^The data in the last two columns represent the *Z* value and the *P* value; ^c^Others: Triple antibiotic use or use of third line antibiotics such as carbapenems. MPP: Mycoplasma pneumoniae pneumonia.

**Table 2:** Comparison of laboratory test results in pediatric MPP with and without respiratory failure

**Laboratory findings**	**MPP with respiratory failure**	**MPP without respiratory failure**	***Z*-score**	***P* value**
*Complete blood count*				
WBC, *10ˆ9/L	9.98 (8.28)	8.09 (5.25)	−6.526	<0.001
Monocyte, %	6.60 (3.25)	7.00 (3.00)	−2.726	0.006
Neutrophil, %	66.50 (20.75)	63.40 (16.75)	−3.571	<0.001
Lymphocyte, %	24.90 (18.45)	26.90 (15.30)	−3.190	0.001
Hemoglobin, g/L	123 (13)	125 (14)	−3.695	<0.001
Platelet, *10ˆ9/L	337 (231.5)	295 (173.25)	−3.311	0.001
C-reactive protein, mg/L	20.97 (28.58)	15.90 (19.60)	−5.030	<0.001
*Inflammatory immune indicators*				
Interleukin-2, pg/mL	1.9 (0.7)	1.9 (0.9)	−0.444	0.657
Interleukin-4, pg/mL	2.3 (1.0)	2.4 (1.0)	−0.580	0.562
Interleukin-6, pg/mL	36.7 (66.9)	28.3 (40.7)	−3.440	0.001
Interleukin-10, pg/mL	9.7 (9.4)	7.1 (4.3)	−8.065	<0.001
Interferon-α, pg/mL	2.0 (1.1)	2.0 (1.4)	−0.659	0.510
Interferon-γ, pg/mL	7.9 (19.6)	5.8 (9.1)	−3.733	<0.001
Immunoglobulin G, g/mL	9.6 (3.3)	9.8 (3.1)	−1.524	0.127
Immunoglobulin A, g/mL	1.21 (0.93)	1.29 (0.86)	0.033	0.857
Immunoglobulin M, g/mL	1.4 (0.8)	1.3 (0.7)	−2.000	0.046
Complement component 3, g/mL	1.3 (0.4)	1.3 (0.3)	−2.450	0.014
Complement component 4, g/mL	0.44 (0.21)	0.48 (0.17)	3.487	0.062
Retinol binding protein, mg/mL	17.0 (8.4)	20.1 (7.1)	−1.335	0.182
Immunoglobulin E, IU/mL	140 (358)	88 (247)	−2.88	0.004
Procalcitonin, ng/mL	0.11 (0.22)	0.09 (0.11)	−4.846	<0.001
Erythrocyte sedimentation rate, mm/H	29.88 (26.42)	27.04 (20.53)	−2.102	0.036
*Coagulation function indicators*				
International Normalized Ratio	1.06 (0.15)	1.07 (0.13)	13.382	0.337
Fibrinogen, g/L	3.98 (1.42)	4.02 (1.13)	−1.746	0.081
Activated partial thromboplastin time, s	28.9 (4.0)	28.1 (3.2)	−4.013	<0.001
Thrombin time, s	17.5 (1.7)	17.4 (1.5)	−2.407	0.016
D-dimer, mg/L	1.33 (2.42)	0.47 (0.36)	−16.264	<0.001
*Biochemical indicators*				
Direct bilirubin, Umol/L	1.4 (0.7)	1.4 (0.7)	−1.77	0.077
Indirect bilirubin, Umol/L	4.7 (2.6)	4.3 (2.2)	−3.352	0.001
Alanine aminotransferase, U/L	16 (15)	14 (8)	−5.01	<0.001
Aspartate aminotransferase, U/L	38 (30)	35 (19)	−4.476	<0.001
Total bile acids, Umol/L	5.3 (5.0)	4.9 (4.6)	−1.265	0.206
Lactate dehydrogenase, U/L	410 (319)	341 (224)	−5.797	<0.001
Creatine kinase, U/L	100 (109)	100 (77)	−0.409	0.683
Creatine kinase-MB isoenzyme, U/L	22 (25)	21 (26)	−0.085	0.932

Values are presented as median (interquartile range) as applicable. MPP: Mycoplasma pneumoniae pneumonia.

Complication statistics revealed higher incidences of pulmonary consolidation (29.22%) and pleural effusion (21.40%) in children with MPP. Extrapulmonary complications included liver function impairment (4.99%), myocardial injury (1.39%), gastrointestinal issues (18.47%), nonspecific rash (12.54%), and neurological complications (1.06%). Subgroup analysis indicated that children with MPP and respiratory failure had significantly higher rates of pulmonary consolidation, pleural effusion, liver function impairment, and myocardial injury (*P* < 0.05). Table 2 summarizes laboratory findings for both groups. Blood tests showed that children with MPP and respiratory failure had significantly higher levels of WBC, neutrophils, PLT, and CRP compared to those without respiratory failure (*P* < 0.05). Additionally, this group exhibited lower monocyte and lymphocyte percentages as well as reduced hemoglobin (HB) levels (*P* < 0.05). Elevated inflammatory and immune markers—including IL-6, IL-10, IFN-γ, IgM, IgE, PCT, and ESR—were observed in children with respiratory failure (*P* < 0.05), whereas C3 levels were significantly lower (*P* < 0.05). No significant differences were found in IL-2, IL-4, TNF-α, IgG, IgA, C4, or RBP levels (*P* > 0.05). Coagulation tests revealed increased INR, APTT, TT, and D-dimer levels in children with respiratory failure (*P* < 0.05), while Fib levels showed no significant difference (*P* > 0.05). Given the heightened risk of hepatic and myocardial injury in MPP, hepatic and cardiac function parameters were evaluated. Children with respiratory failure displayed significantly higher levels of indirect bilirubin (IBIL), alanine aminotransferase (ALT), aspartate aminotransferase (AST), and LDH (*P* < 0.05). However, no significant differences were observed in direct bilirubin (DBIL), total bile acid (TBA), creatine kinase (CK), or CK-MB levels (*P* > 0.05).

### Risk factors of respiratory failure

**Figure 2.**
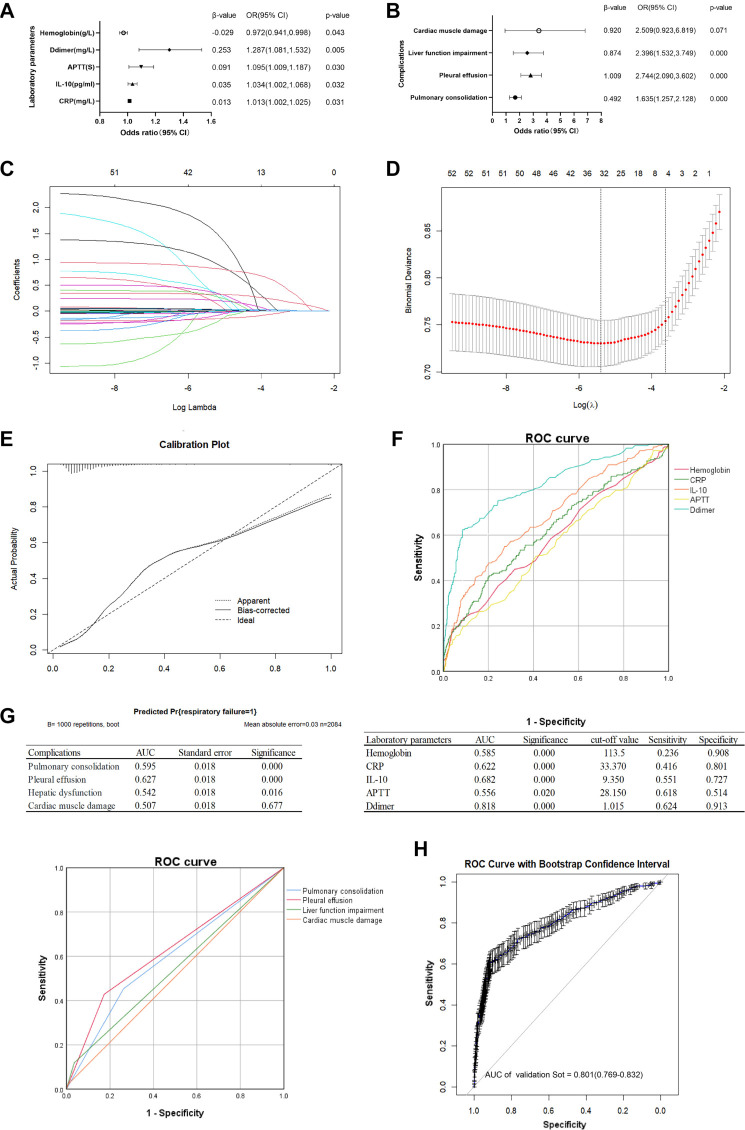
**Identification of high-risk factors for respiratory failure in children with MPP.** (A and B) Forest plot of logistic regression analysis on the association between clinical laboratory indicators and disease complications with respiratory failure in children with MPP. (C) The Lasso coefficient curves for clinical laboratory indicators and disease complications. Draw a vertical line at the value selected by 10-fold cross-validation. As the λ value decreases, the degree of model compression increases, enhancing the model’s ability to select significant variables. (D) Cross-validation results. The range between the two dashed lines represents the positive and negative standard deviation of log(λ). The dashed line on the left indicates the value of log(λ) for the harmonic parameter when the model error is minimized. When log(λ) ═ −3.625, four variables are selected. (E) The calibration plot represents the predicted probability and the actual incidence of respiratory failure in children with MPP. (F and G) ROC and AUC for the predictive model, clinical laboratory indicators, and disease complications. (H) Bootstrap resampling of 1000 replications for internal validity. MPP: Mycoplasma pneumoniae pneumonia; Lasso: Least Absolute Shrinkage and Selection Operator; AUC: Area under the curve.

Subsequently, a multivariable analysis was performed on the clinical indicators identified through univariate analysis. The Hosmer–Lemeshow test yielded a *P* value of 0.359, indicating good model fit and high consistency. Significant predictors of respiratory failure in children with MPP included CRP (OR ═ 1.013, 95% CI ═ 1.002–1.025), IL-10 (OR ═ 1.034, 95% CI ═ 1.002–1.068), APTT (OR ═ 1.095, 95% CI ═ 1.009–1.187), D-dimer (OR ═ 1.287, 95% CI ═ 1.081–1.532), and HB (OR ═ 0.972, 95% CI ═ 0.941–0.998) (*P* < 0.05) (Figure 2A). In the analysis of complications, the Hosmer–Lemeshow test yielded a *P* value of 0.249, indicating adequate model fit. Significant predictors included pulmonary consolidation (OR ═ 1.635, 95% CI ═ 1.257–2.128), pleural effusion (OR ═ 2.744, 95% CI ═ 2.090–3.602), liver function impairment (OR ═ 2.396, 95% CI ═ 1.532–3.749), and myocardial injury (OR ═ 2.509, 95% CI ═ 0.923–6.819) (*P* < 0.05). Other indicators, such as WBC, platelets, immune markers, and liver function parameters, were not significant (*P* > 0.05) (Figure 2B). A Lasso logistic regression model was used with cross-validation to construct a risk factor classifier. Six risk factors were selected: age (coefficient ═ 0.955), WBC (1.026), D-dimer (1.255), IgM (1.003), pulmonary necrosis (1.056), and pleural effusion (1.794) (Figure 2C and 2D). In this study, a simple random resampling method without replacement was applied. Samples of the same size were randomly drawn 1000 times to generate bootstrap samples. These bootstrap samples were then used to calculate statistical estimates and assess the variability and uncertainty of the results. A calibration plot based on 1000 bootstrapped resamples demonstrated high consistency between the predicted and observed probabilities of respiratory failure in children with MPP (Figure 2E).

### Model validation and ROC for respiratory failure

ROC analysis of variables associated with respiratory failure complicating MPP demonstrated that CRP, IL-10, APTT, D-dimer, and HB were statistically significant predictors of respiratory failure (*P* < 0.05). Among these, the D-dimer ROC curve showed the largest AUC of 0.818, with an optimal cutoff value of 1.105 mg/L. The sensitivity and specificity for predicting respiratory failure in MPP were 62.4% and 91.3%, respectively (Figure 2F). Statistical analysis of complications identified pulmonary consolidation, atelectasis, pleural effusion, and liver function impairment as significant predictors of respiratory failure in children with MPP (*P* < 0.05). Among these, pleural effusion had the highest AUC of 0.627. Although hospitalization duration and pleural effusion demonstrated predictive capabilities, their performance was inferior to that of D-dimer in predicting respiratory failure in children with MPP (Figure 2G). Internal validation of the D-dimer disease prediction model using bootstrap analysis with 100 bootstrap samples yielded similar results, with an AUC of 0.801 (95% CI: 0.769–0.832) (Figure 2H).

### D-dimer and clinical indicators correlation

**Figure 3.**
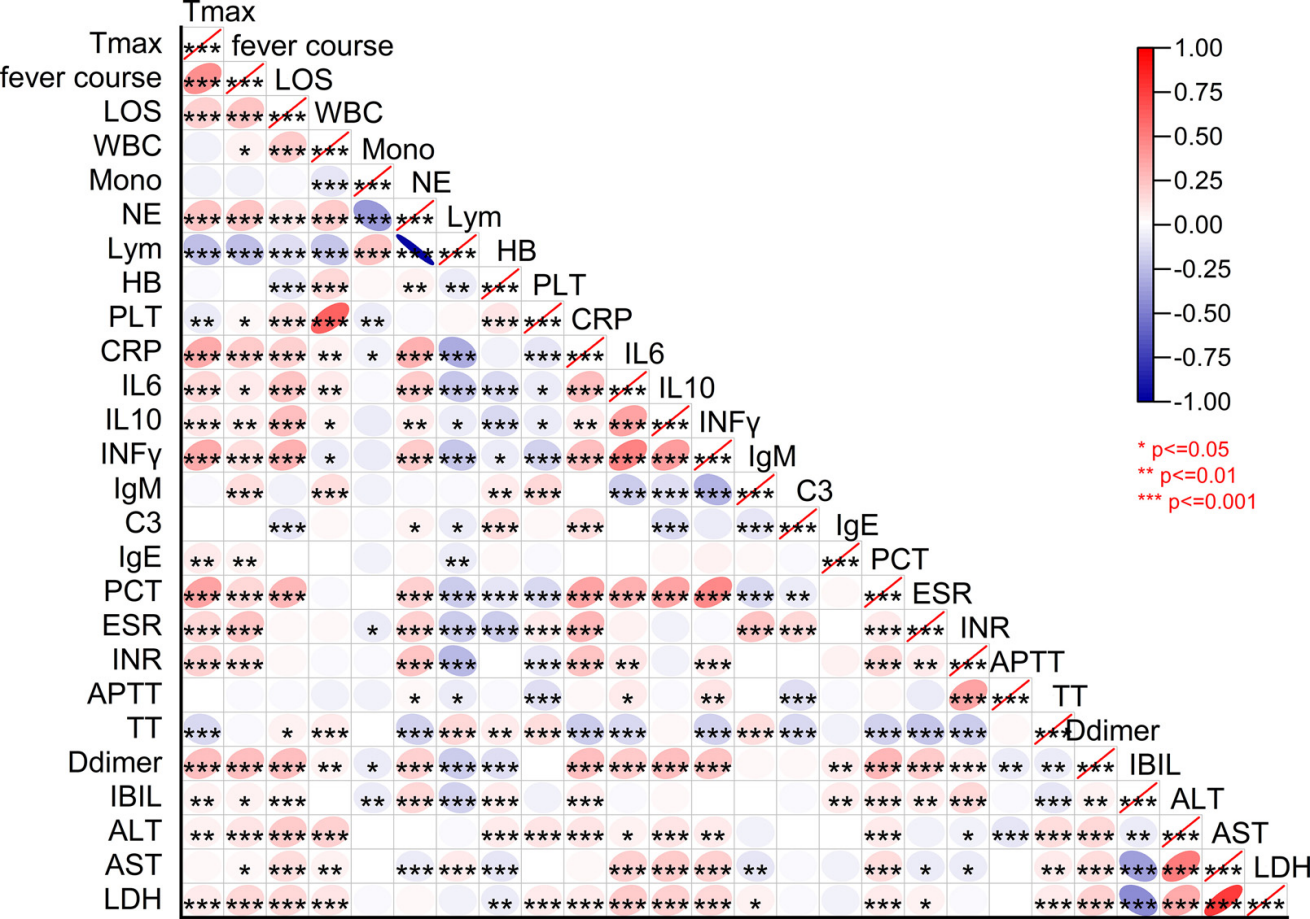
**Heatmap analysis of the correlation between clinical symptoms and clinical indicators in children with MPP.** MPP: Mycoplasma pneumoniae pneumonia; LOS: Length of hospital stay; WBC: White blood cell count; HB: Hemoglobin; ALT: Alanine aminotransferase; AST: Aspartate aminotransferase; LDH: Lactate dehydrogenase; IBIL: Indirect bilirubin; ESR: Erythrocyte sedimentation rate; PCT: Procalcitonin; CRP: C-reactive protein.

To clarify the role of D-dimer in MPP, and considering that the majority of clinical data in this study were not normally distributed, a Spearman rank correlation analysis was performed to evaluate the relationship between D-dimer levels and various clinical indicators. The analysis revealed that D-dimer is positively correlated with peak fever (*r* ═ 0.261), duration of fever (*r* ═ 0.269), duration of hospitalization (*r* ═ 0.265), WBC (*r* ═ 0.071), neutrophil ratio (*r* ═ 0.193), CRP (*r* ═ 0.268), IL-6 (*r* ═ 0.212), IL-10 (*r* ═ 0.265), IFN-γ (*r* ═ 0.241), PCT (*r* ═ 0.299), ESR (*r* ═ 0.223), ALT (*r* ═ 0.162), AST (*r* ═ 0.141), and LDH (*r* ═ 0.198). In contrast, D-dimer is negatively correlated with lymphocyte ratio (*r* ═ −0.191) and HB (*r* ═ −0.102) (*P* < 0.05) (Figure 3).

## Discussion

MP infection is highly prevalent and clinically significant, with disease severity ranging from mild to life-threatening. Despite its often benign and self-limiting nature, MP infection is frequently underestimated [4]. Mycoplasma is primarily a mucosal pathogen that parasitizes host epithelial cells, typically targeting the respiratory or urogenital tracts. It exclusively infects humans and spreads through close contact or respiratory droplets [18]. The latency period ranges from 1 to 3 weeks. MP infections occur across all age groups worldwide, with climate, seasonality, and geographical factors generally considered insignificant. However, this study found MP infections to be more common in autumn and winter. Clinical symptoms of MP infection are diverse, with fever and cough being the most common. The cough often manifests as progressive tracheobronchitis and initially presents alongside upper respiratory congestion, flu-like symptoms, and pharyngitis. If the infection progresses to pneumonia, fever typically follows. In severe or fulminant cases, MP infection can be life-threatening [19]. Severe respiratory symptoms may lead to decreased blood oxygen saturation and increased work of breathing, often necessitating hospitalization [20]. Acute respiratory failure caused by MP infection is a critical condition. Post-discharge complications are common in children with acute respiratory failure and are often linked to factors experienced during their critical illness [21]. In this study, respiratory failure accounted for 15.8% of MPP cases. Respiratory failure is characterized by pulmonary mechanical or functional abnormalities, impaired gas exchange, and pulmonary circulation disorders. These issues can prevent the respiratory system from meeting the body’s oxygenation, ventilation, or metabolic demands, potentially resulting in extrapulmonary organ failure, additional complications, or even death [22]. Current consensus suggests that MP infection leading to respiratory failure involves several mechanisms. MP infection can trigger alveolar inflammation, causing lung tissue damage and impairing oxygen and carbon dioxide exchange. It may also induce airway obstruction, which hinders gas flow and results in breathing difficulties. Furthermore, MP infection can create conditions conducive to secondary infections by other bacteria or viruses [23, 24]. Children with severe cases require intensive care, aggressive respiratory support, and treatment targeting the underlying disease to improve their chances of recovery [25]. In summary, early identification of respiratory failure is crucial in children with MPP for several reasons. First, it enables the timely implementation of appropriate therapeutic measures, particularly for severe cases that may require interventions such as mechanical ventilation. Second, it aids in predicting prognosis, as respiratory failure often indicates greater disease severity. Children with severe respiratory failure typically have poorer prognoses and may require prolonged treatment and rehabilitation. Lastly, early recognition and management of respiratory failure can effectively prevent some complications, improving overall outcomes for affected children.

The treatment of respiratory failure involves airway clearance through suctioning, bronchodilator use, tracheostomy, and endotracheal intubation with respiratory support. Additionally, the management plan includes antibiotics for infections, anticoagulants for pulmonary thromboembolism, and electrolyte supplementation to address fluid imbalances [25]. In this study, children with MPP complicated by respiratory failure showed a significantly higher frequency of steroid use, triple or third-line antibiotics, and the need for fiberoptic bronchoscopy. Respiratory failure is prevalent among pediatric patients, contributing substantially to mortality, disability, and the consumption of healthcare resources [26]. Despite growing awareness of the link between severe MPP and respiratory failure, large-scale clinical studies—particularly randomized controlled trials—remain scarce. To our knowledge, this is one of the few studies investigating the risk factors for respiratory failure in children caused by MP infection. Analysis of inflammatory markers and liver and kidney function tests in different groups of children with MPP revealed that those with respiratory failure had significantly elevated levels of white blood cells, neutrophil ratio, PCT, ESR, CRP, IL-6, IL-10, IFN-γ, ALT, AST, and LDH. Notably, children with MPP complicated by respiratory failure also experienced significantly longer hospital stays due to the need for intensive and prolonged medical interventions. This highlights a more severe inflammatory response and greater organ function damage in these patients. LDH and CRP levels, in particular, appear to correlate with respiratory function (PaO_2_/FiO_2_) and are established predictors of respiratory failure in COVID-19 patients. These markers could aid in the early identification of patients requiring closer respiratory monitoring and more aggressive treatment to improve outcomes [27]. Similarly, neutrophil ratio and IFN-γ levels may help diagnose severe MPP and predict complications in children [28, 29]. Additionally, this study identified significantly elevated platelet counts in children with respiratory failure. In COVID-19 patients with respiratory failure, enhanced platelet activation has been linked to disease severity, myocardial injury, and mortality [30]. Analysis of complications also revealed that the proportion of children with respiratory failure who developed pleural effusion was significantly higher, which may hold clinical value in predicting respiratory failure. Previous research has confirmed that pleural effusion is associated with a more severe clinical course, poorer treatment responses, and severe pneumonia based on the extent of lung lesions [31]. Further findings from our data analysis indicate that children with MPP complicated by respiratory failure exhibited higher IgE levels, consistent with prior studies. Animal experiments have shown that MP produces the Community-Acquired Respiratory Distress Syndrome (CARDS) toxin, which induces excessive mucus secretion, increased Th2 responses, eosinophilia, airway hyperresponsiveness, and elevated IgE levels. These factors contribute to exacerbated asthma symptoms and wheezing [32, 33]. Clinical research has also confirmed that patients with complications, such as necrotizing pneumonia, pneumothorax, rash, obliterative bronchiolitis, or MP-related extrapulmonary diseases exhibit relatively higher IgE levels [34]. Furthermore, MPP patients with elevated IgE levels tend to experience more severe disease, clinical symptoms, and complications [35, 36].

Based on this study, D-dimer has emerged as a crucial risk factor for predicting respiratory failure in children with MPP. D-dimer is a specific fibrin degradation product generated during fibrin monomer activation via cross-linking factors. It serves as a key marker of the fibrinolytic system and an indicator for monitoring inflammation and severe infections [28]. In children with MPP complicated by respiratory failure, a series of pathophysiological changes are frequently observed, including local hypoxia, ischemia, and acidosis. The direct invasion of MP pathogens and their toxins triggers a cascade of events, activating the coagulation, fibrinolysis, kinin, and complement systems. This activation disrupts the body’s physiological balance, leading to coagulation dysfunction [37]. A hallmark of this dysfunction is the development of a hypercoagulable state, where excessive blood clotting occurs. To counteract this, fibrin degradation intensifies, resulting in elevated D-dimer levels [38]. Moreover, MP stimulates inflammatory cells, such as macrophages and neutrophils, prompting the release of inflammatory mediators like TNF-α and IL-6 [39, 40]. These mediators can damage vascular endothelial cells, further exacerbating the condition. Damaged endothelial cells expose subendothelial collagen fibers, activating the intrinsic coagulation pathway, increasing fibrin production, and triggering fibrinolysis, which elevates D-dimer levels. Additionally, endothelial damage disrupts vascular tone and anticoagulation functions, impairing blood circulation and gas exchange in the lungs. This creates a vicious cycle that worsens respiratory function and accelerates respiratory failure progression [41, 42]. Our study also found that D-dimer correlates significantly with clinical indicators of MPP in children, particularly inflammatory markers, consistent with previous research [43]. Elevated D-dimer levels have clinical significance in predicting complications like necrotizing pneumonia [44], refractory MPP [45], and poor prognosis in children. Children with high D-dimer levels often experience complications, such as pleural effusion, myocardial damage, and liver damage [46]. They also present more severe symptoms and require longer treatment durations [43]. Notably, elevated D-dimer levels are associated with worse outcomes in respiratory diseases. Studies on patients with pneumonia and COPD have shown that higher D-dimer levels correlate with lower survival rates and an increased risk of death from respiratory failure in the general population [38]. In conclusion, D-dimer is a critical marker for assessing disease severity, predicting complications, and forecasting the prognosis of respiratory failure in children with MPP. Its role underscores the complex interplay between inflammation, coagulation, and respiratory dysfunction in these patients.

This study has several limitations. The sample source is relatively restricted, as the children were recruited from a tertiary children’s hospital. This setting may overrepresent severe infection cases, which limits the generalizability of the findings to milder cases typically encountered in primary or secondary care. Pathogen detection also presents challenges: false negatives make it impossible to rule out concurrent infections with other pathogens, and the diagnostic methods employed may have overlooked some pathogens. These limitations could affect the study’s ability to fully understand disease etiology and the relationships between contributing factors. Additionally, the relatively short follow-up period only permitted the observation of immediate and short-term outcomes. As a result, the study is unable to capture the long-term consequences of the disease, which restricts a comprehensive assessment of its full impact on patients’ health and development.

## Conclusion

An elevated D-dimer shows a notable positive correlation with inflammatory markers, liver function, and LDH in children suffering from MPP. Serum D-dimer levels exceeding 1.015 mg/L may be associated with an increased risk of respiratory failure in these children. Therefore, during the clinical diagnosis and treatment of MPP, greater emphasis should be placed on monitoring coagulation function, with particular attention to serum D-dimer levels. For MPP patients with elevated D-dimer levels, clinicians should closely observe the child’s respiratory condition and consider implementing appropriate respiratory management strategies to help reduce the risk of respiratory failure. However, due to the limitations of this study, further research is essential to validate and expand upon these findings.

## Data Availability

The datasets used and/or analysed during the current study available from the corresponding author on reasonable request.
